# Prevalence of *Toxoplasma gondii *Antibodies and DNA in Iranian HIV Patients

**DOI:** 10.30699/IJP.14.1.68

**Published:** 2018-12-27

**Authors:** Anahita Bavand, Arezoo Aghakhani, Minoo Mohraz, Mohammad Banifazl, Afsaneh Karami, Majid Golkar, Jalal Babaie, Parviz Saleh, Setareh Mamishi, Amitis Ramezani

**Affiliations:** 1 *MSc, Dept. of Clinical Research, Pasteur Institute of Iran, Tehran, Iran*; 2 *Anatomical & Clinical Pathologist, Dept. of Clinical Research, Pasteur Institute of Iran, Tehran, Iran*; 3 *Infectious Diseases Specialist, Iranian Research Center for HIV/AIDS, Tehran, Iran*; 4 *Pediatrician, Iranian Society for Support of Patients with Infectious Disease, Tehran, Iran*; 5 *Infectious Diseases Specialist, Zanjan University of Medical Sciences, Zanjan, Iran*; 6 *PhD, Molecular Parasitology Laboratory, Dept. of Parasitology, Pasteur Institute of Iran, Tehran, Iran*; 7 *Infectious Diseases Specialist, Kidney Research Center, Tabriz University of Medical Sciences, Tabriz, Iran*; 8 *Pediatrician, Pediatric Infectious Disease Research Center, Tehran University of Medical Sciences, Tehran, Iran*; 9 *Infectious Diseases Specialist, Dept. of Clinical Research, Pasteur Institute of Iran, Tehran, Iran*

**Keywords:** Human Immunodeficiency Virus (HIV), IgG, IgM, Toxoplasma gondii, Reactive Inhibition Antibodies

## Abstract

**Background & Objective:**

*Toxoplasma gondii *infection has public health importance and can lead to serious diseases in immunosuppressed patients, such as HIV cases. Appropriate control of *T. gondii *infection in HIV patients requires information about the prevalence of *T. gondii *antibodies and DNA in different population. In this study, we aimed to determine the prevalence of *Toxoplasma gondii *antibodies and DNA in HIV patients in Tehran, Iran.

**Methods:**

A total of 149 HIV patients from the Iranian Research Center for HIV/AIDS, Tehran, Iran were enrolled in the study. Anti-Toxoplasma IgG and IgM were detected by ELISA and *T. gondii *DNA was evaluated by PCR and quantita- tive real-time PCR. IgG positive samples were also assessed for their avidity.

**Results:**

Anti-Toxoplasma IgG and IgM were positive in 46.3% and 2.7% of cases respectively. 92.7% of our patients showed past infection and 4.3% revealed recently acquired toxoplasmosis based on their IgG avidity test. T. gondii DNA was not detected by PCR but real-time PCR results showed DNA in 4.7% of total patients and 13.1% of the IgG seropositive cases.

**Conclusion:**

Our findings indicated that latent toxoplasmosis was relatively prevalent in our study population, but new

*T. gondii *infection had low prevalence. Almost half of our patients were IgG negative and at risk of acquiring toxoplasma infection. Low copy numbers of DNA were detected in 4.7% of the cases without any clinical manifestation. Therefore, detection and monitoring of anti-Toxoplasma antibodies and DNA in HIV patients is substantial to estimate the risk of reactivation and new infection.

## Introduction

Toxoplasmosis is a parasitic disease caused by *Toxoplasma gondii (T. gondii)*, an obligate intracellular parasite. It has a wide distribution and it affects almost 30% of the world’s population, with a variable prevalence in dif- ferent countries (1).

In the majority of immunocompetent individuals, *T. gondii *infection is a subclinical and asymptomatic diseasewhich may lead to latent infection characterized by the persistence of the organism within host tissues without any symptoms (2). However *T. gondii *can lead to acute and life-threatening disease in immunocompromised people such as HIV individuals, cases with malignancy and subjects receiving organ transplantation (3, 4). *T. gondii *infection is considered as one of the most important opportunistic infections in patients with HIV, and is also considered as the main cause of morbidity and mortality in these patients (5, 6).

The prevalence of *T. gondii *infection in HIV patients varies throughout the world. The overall seroprevalence rate reported is 35.8% but based on the region it differs from 60.7% in the Middle East and North Africa to reach 49.1% in Latin America, 44.9% in sub-Saharan Africa, 30.1% in western and central Europe and North America while it is 25.1% in Asia and the Pacific (7).

The main cause of toxoplasmosis in an HIV patient is the reactivation of latent infection particularly in the brain, leading to toxoplasmic encephalitis (TE), especially when CD4 count reduces to less than 100 cells/mm3 (7). Patients with toxoplasmosis and HIV co-infection have 30 to 40% risk of TE (8). This is a fatal infection in HIV patients without receiving antiretroviral therapy (9).

Serological tests are used for the diagnosis of toxoplasmosis, but these tests are not reliable in immunocom- promised patients due to suppression of the immune system. Molecular tests are now considered as important method for the diagnosis of toxoplasma infection in immunosuppressed individuals, especially when serologi- cal techniques fail (10). Some authors mentioned the association of *T. gondii *IgG antibodies and DNA with the reactivation of toxoplasmosis and its clinical manifestation in HIV patients (11, 12).

However, appropriate diagnosis and control of *T. gondii *infection in HIV patients require adequate informa- tion regarding the prevalence of *T. gondii *antibodies and DNA in different population. Due to limited data on molecular diagnosis of Toxoplasma infection and its association to *T. gondii *antibodies in Iranian HIV patients, we aimed to determine the prevalence of *Toxoplasma gondii *antibodies and DNA in HIV patients in Tehran, Iran.

## Materials and Methods

**Study Population**

In this cross-sectional study, 149 HIV patients who were referred to the Iranian Research Center for HIV/AIDS in Tehran, Iran were consecutively enrolled from May to September 2017. The study protocol was approved by the Pasteur Institute of Iran Ethical Committee and informed consent was obtained from subjects prior to the study.

CD4 count was determined by flowcytometry and defined as cells/mm3.

**Detection of Anti-Toxoplasma IgG and IgM Antibodies**

All plasma samples were screened using the standard enzyme-linked immunosorbent assay (ELISA) com- mercial kits for the detection of anti-Toxoplasma IgG and IgM antibodies (EUROIMMUN, Lubek, Germany) in accordance with the manufacturer’s instructions.

**Assessment of IgG Avidity**

For differentiation between recently acquired and past infection of T. gondii infection, anti-Toxoplasma IgG positive specimens were subjected for IgG avidity test using ELISA kit (EUROIMMUN, Lubek, Germany). A result of ˂40% was interpreted as low avidity (T. gondii infection was acquired within the last 3 months) be- tween 40-60% as equivocal range and ˃60% as high avidity (Toxoplasma infection was acquired more than 3 months ago).

**DNA Extraction and Polymerase Chain Reaction**

Toxoplasma-DNA was extracted from 200μl of whole blood using QIAamp® DNA Mini Kit (QIAGEN,

Hilden, Germany) according to manufacturer’s instructions.

In order to evaluate the suitability of the extracted DNA, β-globin gene amplification was performed using PCO3 (5’-ACACAACTGTGTTCACTAGC-3’) and PCO4 (5’- CAACTTCATCCACGTTCACC-3’) primers which amplify a 110-bp fragment. PCR was carried out in a 25μl amplification mixture containing 1μl of ex- tracted DNA, 1.5 mM MgCl2 , 15 mM Tris-HCl (pH 8.0), 0.2 mM dNTP, 50 mM KCl, 10 pmol of each of prim- ers and 1.5 U Taq polymerase (YTA PCR Master Mix, Iran). β-globin positive samples were subjected to PCR.

Polymerase chain reaction was carried out using primer pair TOXO1 (CGCTGCAGGGAGGAAGAC- GAAAGTTG) and TOXO2 (CGCTGCAGACACAGTGCATCTGGATT) which were selected from the 50 and 30 end of the 529 bp fragment (RE) respectively. The PCR reaction was performed in a 25 ml reaction mixture containing 15 mM Tris-HCl (pH 8), 50 mM KCl, 1.5 mM MgCl2, 0.2 mM dNTP, 10 pmol of each primer and 1.5 U Taq polymerase (YTA PCR Master Mix, Iran). Amplification was performed as initial 7 minute (min) dena- turation at 94°C, followed by 35 cycles of amplification including denaturation for 1 min at 94°C, annealing for 1 min at 55°C and extension for 1 min at 72°C. Strand synthesis was completed at 72°C for 10 min and stored in 4°C for 5 min. The expected size of the PCR product was 529 bp. Each batch included negative control contain- ing water and extracted DNA from *T. gondii *tachyzoites RH-strain as positive control.

The PCR products were electrophoresed on a 1.5% agarose gel with the 100-bp DNA ladder (Sinaclon, Iran) and stained with DNA stain and visualized by ultraviolet transillumination.

**Quantitative Real Time PCR Assays**

Quantitative Real time PCR Assays for REP-529 was performed according to the method previously described by Babaie et al. (13). Real time PCR was conducted using a BIO‐RAD DNA Engine Thermal Cycler under the following conditions: an initial denaturation at 95 °C for 10 min, followed by 35 cycles of 30 sec at 95 °C, 30 sec at 58 °C and 30 sec at 72 °C. The final extension step was set for 20 min at 72 °C. The analytical detection limit of assay is 10 copies per PCR reaction (25µl).

**Statistical Analysis**

Statistical analyses were conducted using SPSS statistics software (version 16, Chicago, IL, USA). The Chi square test or Fisher’s exact test was used to compare variables. Data is presented as mean ± SD or, when indi- cated, as an absolute number and percentage. *P*-values <0.05 were considered statistically significant.

## Results

A total of 149 HIV infected patients between the ages of 18 to 74 years (mean age of 39.52 ± 8.07) including 81.2% male and 18.8% female were enrolled in the study. The most possible routes of HIV transmission were intravenous drug use (17.2%), heterosexual contact (27.6%), infected blood and blood products (2.8%), vertical transmission (0.7%), homosexual (2.8%), tattoo (1.4%), IDU and heterosexual (7.6%), IDU and tattoo (6.9%), heterosexual and tattoo (0.7%), homosexual and heterosexual (0.7%), IDU and heterosexual and tattoo (20%), IDU and heterosexual and blood transfusion (0.7%), IDU and heterosexual and homosexual (1.4%) and in 9.7% the route of HIV acquisition was not identified. The mean CD4 count was 458.08± 268.267 cells/mm3. 78.5% of patients receiving highly active antiretroviral therapy (HAART).

2.7% (4/149) of the cases were positive for anti-Toxoplasma IgM and 46.3% (69/149) were positive for IgG antibodies, of which 64 (92.7%) cases showed past infection and 3 (4.3%) patients revealed recently acquired toxoplasma infection according to their IgG avidity test. 2 (2.9%) subjects demonstrated equivocal IgG avidity range. 53.7% of our HIV cases were IgG negative and at risk of acquiring Toxoplasma infection.

Three out of four IgM positive cases had IgG simultaneously and all 3 cases had low IgG avidity with CD4 count ≥500 cells/mm3, only one subject was IgM positive.

All cases were categorized in four age groups as: 18-28, 29-39, 40-49, and ≥ 50 years. The highest (49.3%) patients and the lowest (33.3%) IgG antibody rates were found within the age groups of 40-49 and 18-28 years old, re- spectively (table 1). The IgG seroprevalence was slightly higher in females (50%) than in males (45.5%) (Not significant).

**Table 1:** Frequency of Toxoplasma DNA and antibodies based on age in HIV positive

**Age groups**	**N (%)**	**Anti -Toxoplasma IgM**	**Anti-Toxoplasma IgG**	***T. gondii *** **DNA**
18-28	9 (6%)	0(0%)	3 (33.3%)	0(0%)
29-39	74 (49.7%)	1 (0.7%)	26 (35.1%)	12 (8.1%)
40-49	53 (35.6%)	3 (2%)	34 (49.3%)	7 (4.7%)
50≤	13 (8.7%)	0(0%)	6 (46.2%)	0(0%)

Human immunodeficiency virus (HIV)

*T. gondii *DNA was not detected by PCR, but quantitative real-time PCR results showed *T. gondii *DNA in 7 (4.7%) patients. The titers of *T. gondi*i DNA was from 10 to 28.39 copies per reaction (25µl). From seven DNA positive samples, 4 cases had IgG simultaneously with high IgG avidity. One case had IgM, IgG and DNA concurrently with low IgG avidity (probably ongoing infection) and only 2 subjects were DNA positive. Out of seven DNA positive cases, six subjects were injecting drug users with a past history of incarceration.

From 69 IgG positive cases, *T. gond*ii DNA was detected in 5 (13.1%) patients. Furthermore, 2.5% (2/80) of serology negative patients showed positive PCR results.

**Table 2:** Rate of *Toxoplasma gondii *antibodies and DNA based of CD4 counts in our cohort of study

**CD4 count (cells/mm** **3** **)**	**0-99** **N (%)**	**100-199** **N (%)**	**200-499** **N (%)**	**500≤ N (%)**
Total of patients	6 (4.1%)	11 (7.6%)	75 (51.7%)	53 (36.6%)
Anti-Toxoplasma IgM positive patients	0 (0%)	0 (0%)	0 (0%)	4 (100%)
Anti-Toxoplasma IgG positive patients	3 (50%)	4 (36.4%)	38 (50.7%)	23 (43.4%)
DNA positive patients	0 (0%)	1 (9.1%)	5 (6.7%)	1 (1.9%)

Human immunodeficiency virus (HIV)

The rate of *Toxoplasma gondii *antibodies and DNA based on CD4 counts were shown in Table 2. There was no significant difference between anti-Toxoplasma antibodies rate and DNA frequency with mean CD4 count. 

## Discussion

This study investigated the prevalence of *Toxoplasma gondii *antibodies and DNA in HIV patients in Tehran, Iran. Anti-Toxoplasma IgG and IgM were positive in 46.3% and 2.7% of the cases respectively and 4.7% of the subjects were positive for *T. gondii *DNA by real time PCR. 92.7% of our patients showed past infection and 4.3% revealed recently acquired toxoplasmosis based on their IgG avidity test.

Co-infection of *T. gondii *and HIV infection is a major public health issue which can affect the course of both infections by the interaction of two pathogens and by suppressing host immune system (14). Almost half of the HIV individuals have co-infection with *T. gondii *(15, 16).

Seroprevalence of Toxoplasma infection varies greatly worldwide due to socioeconomic conditions, cultural habits, different lifestyles, climate, geographical areas, age, residing in rural areas, educational status and having raw or undercooked meat (17). Different studies reported the seroprevalence rate of *T. gondii *in HIV patients from less than 10% to over 90% in different countries and even within one country, this variation is substantial (7). This seroprevalence is also very variant in the different parts of Iran (15, 18-21). Even in Tehran, two dif- ferent seroprevalence rates were reported by Rostami (22) and Mohraz et al. (19.1% and 49.7% retrospectively) (23). Our finding is consistent with Mohraz et al. study but the rates are much higher than what is expressed in Rostami et al. survey regarding *T. gondii *seroprevalence rate in Tehran.

Serologic tests such as IgM and IgG are the main methods for diagnosis of toxoplasmosis but these assays con- stitute some difficulties to differentiate acute form from chronic and the reactivation of infection (24). The detec- tion of anti-*T.gondii *IgM as a routine diagnostic test of toxoplasmosis has some limited value in the management of HIV patients (25). The IgG avidity test is a qualitative assay which distinguishes chronic toxoplasmosis from a recently acquired infection and determines the status of toxoplasma infection. Low IgG avidity suggests acute infection, while high IgG avidity indicates chronic or reactivated infection (26, 27).

Walle et al. found anti-*T.gondii *IgM in 10.7% of HIV individuals (28). A study from India detected 6% anti-*T. gondii *IgM in HIV cases (29). Zeleke et al found toxoplasma IgM antibody in 2.2% of HIV positive women in reproductive age and all IgM positive samples were positive for IgG concurrently (30). They found low rate of *T. gondii *IgM (2.2%) in comparison to IgG (94.4%). Same results in HIV patients reported from Mexico and South Africa (31, 32). We also found low rate of *T. gondii *IgM in comparison to IgG and three from four our IgM posi- tive cases had IgG simultaneously with low IgG avidity. Additionally, an overall 4.3% of our cases had low IgG avidity which indicates recently acquired Toxoplasma infection and such is not common in our cases. This data emphasizes that the reactivation of chronic *T. gondii *infection is the dominant way of acquiring toxoplasmosis in HIV cases that is not a new infection (32, 33, 34). However, incidence of reactivation is associated with the prevalence and concentration of *T. gondii *IgG antibodies (35).

Today, molecular methods are known as important diagnostic tools for the detection of toxoplasmosis in immunosuppressed hosts (10). Some authors mentioned that the detection of *T. gondii *DNA in the blood indicates active infection and is closely associated with clinical manifestation of HIV patients (11,12) while other molecular studies on HIV patients without cerebral toxoplasmosis did not confirm this association. Gashout et al. detected *T.gondii *DNA in 60% of asymptomatic seropositive HIV patients with a CD4 count less than 100 cells/ μl (10). In another study, Toxoplasma DNA was detected in 25% of HIV patients in stages 3 and 4 with positive

*T. gondii *IgG (36). Ayi et al. detected *T. gondii *DNA in 54.7% of HIV seropositive subjects and DNA was found frequently in cases with lower CD4 count (11). In an investigation in Brazil on AIDS patients without cerebral toxoplasmosis, only 2.34% of patients had *T.gondii *DNA (12). Rostami et al. from Iran reported only one posi- tive PCR sample in their HIV cohort (22). In our study, *T. gondii *DNA was observed in 13.1% of the seropositive cases, which is less than several surveys conducted on the same population but is in agreement with Rostami et al. and Colombo et al. surveys (12, 22). However, we enrolled a low number of patients with a CD4 count less than 100 and most of our patients receiving HAART without clinical manifestation of toxoplasmosis. Hence, we should expect a low number of DNA positive cases in this study. Although the presence of Toxoplasma DNA in blood could indicate a recent infection or clinically active disease but reversely, it can be just due to shedding a low amount of parasites from tissue cysts into the blood at a subclinical value, especially in asymptomatic cases which is detectable only by real time PCR (37).

Furthermore, in our study, two patients (2.5%) with negative serology showed positive real time PCR results, but we should consider that negative serology does not exclude the risk of toxoplasmosis reactivation (38, 39, and 40).

## Conclusion

We evaluated the total burden of *T. gondii *infection by measuring *T. gondii *IgG, IgM, IgG avidity test and DNA in Iranian HIV patients. Our data showed that latent toxoplasmosis was relatively prevalent in our study population, but new *T. gondii *infection had low prevalence. However, almost half of our HIV population was IgG negative and at risk of acquiring infection. Low copy numbers of *T. gondii *DNA were detected in 4.7% of the total patients and 13.1% of seropositive cases were without any clinical manifestation. As appropriate control of *T. gondii *infection in HIV patients is very important, the detection and monitoring of anti-Toxoplasma anti- bodies and DNA in HIV patients is substantial to estimate the risk of reactivation and new infection.
